# Activation of the Endothelin System in Chronic Kidney Disease and Kidney Transplant Recipients—Implications for Disease Progression

**DOI:** 10.3390/ijms27135647

**Published:** 2026-06-23

**Authors:** Milena Ściskalska, Magdalena Król-Kulikowska, Julia Grzybowska, Ewa Tabaka, Wiktoria Pabian, Dominika Pisarek, Krzysztof Benc, Magdalena Kuriata-Kordek, Mirosław Banasik, Marta Kepinska

**Affiliations:** 1 Department of Pharmaceutical Biochemistry, Faculty of Pharmacy, Wroclaw Medical University, 50-556 Wroclaw, Poland; magdalena.krol-kulikowska@umw.edu.pl (M.K.-K.); marta.kepinska@umw.edu.pl (M.K.); 2 Department of Nephrology, Transplantation Medicine and Internal Diseases, Institute of Internal Diseases, Wroclaw Medical University, 50-556 Wroclaw, Poland; julia.grzybowska@usk.wroc.pl (J.G.); ewa.tabaka@student.umw.edu.pl (E.T.); wiktoria.pabian@student.umw.edu.pl (W.P.); dominika.pisarek@student.umw.edu.pl (D.P.); krzysztof.benc@umw.edu.pl (K.B.); magdalena.kuriata-kordek@umw.edu.pl (M.K.-K.); miroslaw.banasik@umw.edu.pl (M.B.)

**Keywords:** endothelin-1, endothelin A receptor, anti-ETAR antibodies, SNP rs5370, SNP rs5333

## Abstract

The endothelin system is critical in chronic kidney disease (CKD) pathogenesis. However, the relative contribution of circulating endothelin-related biomarkers versus genetic variability remains unclear, particularly in diabetic nephropathy (DN) and after kidney transplantation (KTx). This study evaluated plasma concentrations of endothelin-1 (ET-1), endothelin A receptor (ETAR), and anti-ETAR antibodies (ETAR-Ab) in healthy controls, diabetic nephropathy (DN) patients, and DN patients after kidney transplantation (post-KTx). The influence of polymorphisms rs5370 (*EDN1*) and rs5333 (*EDNRA*) on endothelin-related parameters was analyzed. Polymorphisms were genotyped via PCR-RFLP, and endothelial-related parameters were determined by ELISA. Significant endothelin system activation was observed in both DN and post-KTx patients. ET-1 and ETAR concentrations were markedly elevated compared to controls, with the highest ET-1 levels detected in the post-KTx group, whereas ETAR-Ab levels were reduced. A sex-specific association for rs5370 was observed in male patients with the TG genotype (a nearly 4.5-fold higher risk of renal replacement therapy than in female patients). In proteinuric DN patients, the TC genotype (rs5333) was associated with elevated ETAR and ETAR-Ab. Endothelin system dysregulation is a prominent and persistent feature of CKD, noted after kidney transplantation. The endothelin activation, observed in transplant patients particularly, highlights the potential clinical relevance of endothelin-related biomarkers and supports the rationale for therapeutic strategies targeting the endothelin pathway, including endothelin receptor antagonists.

## 1. Introduction

Chronic kidney disease (CKD) represents a major global health burden, with diabetic nephropathy (DN) being its leading cause and a primary driver of end-stage renal disease worldwide [1,2,3,4]. Kidney transplantation (KTx) remains the optimal treatment for end-stage disease [3,4]. Several factors are involved in the pathophysiology of DN, including metabolic and hemodynamic alterations, oxidative stress, and the activation of the renin–angiotensin system.

The endothelin system has emerged as a key regulator of vascular and renal function. The endothelin (ET) peptide is a potent vasoconstrictor produced by endothelial cells, podocytes, and mesangial cells [5]. There are three variants of the vasoactive peptide (ET-1, ET-2, ET3), with ET-1 being the predominant subtype in vivo and the most biologically active subtype [5,6]. ET-1 exerts its physiologic effects through two G-protein-coupled receptor (GPCR) subtypes: endothelin receptor A (ETAR) and endothelin receptor B (ETBR) [6,7,8]. The vascular smooth muscle ETA receptor is the primary receptor driving the potent vasoconstrictor effects of ET-1 in healthy vasculature [5]. The signaling cascade involves phospholipase C, inositol triphosphate, and diacylglycerol, leading to an increase in intracellular calcium release. Other signaling pathways include phospholipase D, phospholipase A2, and mitogen-activated protein kinase, contributing to the indirect effects of ET-1, such as cellular migration and proliferation [5]. The dysregulation of the endothelin system, especially ETAR, is closely associated with diabetes and renal diseases, making endothelin receptors promising drug targets for the treatment of these diseases [2,4,7].

The association of pathological processes, such as hypoxia, oxidative stress, and proinflammatory cytokine production, with enhanced ETAR expression was shown in [9,10]. It was proven that ETAR mediates the degradation of the glomerular endothelial surface layer via pathologic crosstalk between activated podocytes and glomerular endothelial cells [9]. ETAR is increased in the glomerular endothelial cells of patients with nephropathy and is associated with podocyte damage and glomerular oxidative stress [11]. ETAR activation, induced by the increased renal production of ET-1 in diabetes, leads to macrophage recruitment in the kidney and to TGF-β production by macrophages. Moreover, it was shown that high glucose levels induce mesangial cell ET-1 production. ETAR mediates the proliferative effects of ET-1 on mesangial cells. It causes the mesangial cell contraction, hypertrophy, and generation of inflammatory mediators, cytokines, and growth factors as well as the production of fibronectin in vitro [12]. ETAR signaling contributes to diabetic nephropathy through mesangial matrix expansion, which changes mesangial architecture, glomerular basement membrane thickening, glomerulosclerosis and podocyte loss [11,12]. In addition to ETAR, NF-κB and β-catenin pathways are also involved in podocytes loss [12]. It seems that genetic variations in the gene-encoding ETAR may affect the affinity of ET-1 for the receptor. However, the clinical and functional significance of genetic alteration in the *EDNRA* gene (e.g., rs6842241; rs4835083; rs4639051; rs5333 and rs5343) in the course of DN has not yet been demonstrated [4].

It has been shown that anti-ETAR antibodies (ETAR-Ab) may activate their target receptors and affect receptor hyperactivation and signaling pathways [13,14]. Pathological processes, especially inflammation, are linked to ETAR ectodomain shedding [15]. Shedding is a key cellular mechanism which controls not only the abundance but also the activation and inactivation of transmembrane proteins. For example, this can occur through the degradation of surface receptors [15]; this involves the release of a protein’s ectodomain from the plasma membrane via protease (sheddase) activity. ETAR adopts the canonical seven-transmembrane fold and G protein recruitment mechanism [7]. Canonical sheddases cleave ETAR, thereby releasing a bioactive peptide from its membrane anchor [7,15].

Genetic variability within the endothelin pathway may influence disease susceptibility and progression. Polymorphisms in the *EDN1* gene (rs5370) and the *EDNRA* gene (rs5333) have been investigated in various cardiovascular and renal conditions [16,17,18,19,20,21]; however, their clinical significance remains unclear.

In recent years, interest in the endothelin system has been growing, especially in the context of novel therapeutic targets. It has been shown that a selective endothelin-A receptor antagonism can be used to reduce proteinuria, blood pressure, and arterial stiffness in chronic proteinuric kidney disease [22]. Moreover, the publication titled “Kidney Disease: Improving Global Outcomes (KDIGO) 2025 Clinical Practice Guideline for the Management of IgA Nephropathy and IgA Vasculitis” introduced significant updates regarding the role of selective endothelin-A receptor antagonists (ERAs) in managing proteinuric kidney diseases. These agents, which target the overactivated endothelin system in kidney disease, are highlighted as crucial components in supportive care for reducing proteinuria and slowing disease [23].

Given these considerations, a comprehensive evaluation of circulating endothelin-related biomarkers may provide further insight into the mechanisms underlying CKD progression and post-transplant outcomes. Therefore, this study aimed to assess the concentrations of ET-1, ETAR, and ETAR-Ab in patients with diabetic nephropathy and kidney transplant recipients. It also aimed to evaluate the impact of rs5370 (*EDN1* gene) and rs5333 (*EDNRA* gene) polymorphisms on these parameters. Additionally, it sought to determine whether endothelin system dysregulation is primarily driven by disease-related factors or genetic predisposition.

## 2. Results

### 2.1. Endothelin System Parameters Across Study Groups

A significant dysregulation of the endothelin system was observed across the study groups. Patients with DN and post-KTx exhibited markedly higher plasma concentrations of ET-1 (*p* = 0.0014 and *p* < 0.0001) and ETAR (*p* < 0.0001 for both comparisons) compared to healthy controls. Notably, ET-1 levels were highest in the post-KTx group, exceeding those observed in DN patients (*p* < 0.0001). Similarly, ETAR concentration was significantly elevated in the post-KTx group compared to DN patients (*p* = 0.0359), indicating persistent activation of the endothelin pathway. In contrast, ETAR-Ab levels were significantly reduced in post-KTx patients compared to controls (*p* < 0.0001). DN patients also demonstrated lower ETAR-Ab levels than controls (*p* = 0.0033), although this reduction was less pronounced (Table 1).

Sex-stratified analyses revealed that the observed alterations in ET-1 and ETAR concentrations were consistent in both males and females. No significant differences between sexes were found within individual study groups (Table S1 in the Supplementary Materials).

### 2.2. Results of Genotyping and Genotype Distribution Across Study Groups

We analyzed the concentration of ET-1, ETAR, and ETAR-Ab with respect to single nucleotide polymorphism (SNP). After genomic DNA of the samples was amplified by PCR, the target 385-bp nucleotide sequences for SNP rs5370 (*EDN1* gene) and 173-bp nucleotide sequences for SNP rs5333 (*EDNRA* gene) could be seen in all samples. The identified genotypes were labeled according to the presence or absence of the enzyme restriction site for SNP rs5370 and SNP rs5333.

For SNP rs5370, the TT genotype is homozygous in the absence of the site (bands at 385-bp), the TG genotype is heterozygous in the presence and absence of the site (bands at 164-, 221-, and 385-bp), and the GG genotype is homozygous in the presence of the site (bands at 164- and 221-bp—Figure S1). For SNP rs5333, the TT genotype is homozygous in the absence of the site (bands at 173-bp), the TC genotype is heterozygous in the presence and absence of the site (bands at 84-, 89-, and 173-bp), and the CC genotype is homozygous in the presence of the site (bands at 84- and 89-bp—Figure S2).

No significant differences in genotype frequency were observed between groups (Table 2). It was noted that the frequency of the TT, TG, and GG genotypes for SNP 5370 in the *EDN1* gene was similar in all analyzed groups, healthy controls, and both groups of CKD patients. It was found that the GG genotype was prevalent among the study population. In the case of SNP rs5333 in the *EDNRA* gene, the TT genotype was most frequent among the study population (Table 2). The prevalence of these genotypes was similar when the group of CKD patients was divided into DN patients and post-KTx patients (DN patients after KTx) (Tables S2 and S3 in the Supplementary Materials).

Due to the low frequency of homozygous minor genotypes (the TT genotype for SNP rs5370 and the CC genotype for SNP rs5333), further analyses were primarily conducted using dominant genetic models.

### 2.3. Endothelin System Parameters in Relation to Genetic Variants for SNP rs5370 in the EDN1 Gene and SNP rs5333 in the EDNRA Gene

An increased ET-1 concentration (more than 14-fold) in post-KTx patients with the GG (*p* = 0.013) and TG genotypes (*p* < 0.0001) for SNP rs5370 compared to healthy controls was observed. An increased ET-1 concentration was shown in DN patients with the GG and TG genotypes compared to healthy controls (*p* < 0.0001 for both comparisons). Moreover, a statistically significant increase in the concentration of this peptide in post-KTx patients compared to DN patients with the GG genotype (*p* < 0.0001) was demonstrated (Table 3).

More than a 3-fold increase in the ETAR concentration in DN patients compared to healthy controls (*p* < 0.0001 and *p* = 0.0006 for individuals with the GG and TG genotypes, respectively) was observed. Similarly, ETAR concentrations were elevated in post-KTx patients with the GG (*p <* 0.0001) and TG (*p* = 0.0248) genotypes in comparison with corresponding healthy controls (Table 3).

The ETAR-Ab levels were lowered in DN patients (*p* = 0.0205) and KTx patients (*p* < 0.0001) with the GG genotype compared to healthy controls with the same genotype (Table 3).

The changes in the concentrations of ET-1, ETAR, and ETAR-Ab between the individuals with the GG and TG genotypes across healthy controls and DN or post-KTx patients were not shown (Table 3).

A significant increase in ET-1 concentration was observed in post-KTx patients compared to healthy controls (*p* < 0.0001 for individuals with the TT and TC genotypes for SNP rs5333). Similar differences in the concentration of this peptide in DN patients compared to healthy controls with the TT (*p* < 0.0001) and TC genotypes (*p* < 0.0001) were noted. Moreover, an increased ET-1 concentration was shown in post-KTx patients compared to DN patients with the TT genotype (*p* = 0.0028) (Table 4).

Similarly to the changes in the ET-1 concentration, the ETAR concentration was changed. An increased ETAR concentration was shown in post-KTx patients compared to healthy controls (*p* < 0.0001 for individuals with the TT and TC genotypes) and in DN patients compared to healthy controls (*p* < 0.0001 for individuals with the TT and TC genotypes). Interestingly, a decreased ETAR concentration in post-KTx patients compared to DN patients with the TT genotype (*p* = 0.0215) was shown (Table 4).

A significantly decreased level of ETAR-Ab was observed in post-KTx patients compared to healthy controls with the TT (*p* < 0.0001) and TC genotypes (*p* = 0.0094). The ETAR-Ab level in post-KTx patients with the TT genotype was also lower compared to DN patients (*p* = 0.0215) (Table 4).

The changes in the concentrations of ET-1, ETAR, and ETAR-Ab between the subjects with the TT and TC genotypes for SNP rs5333 in the *EDNRA* gene, in healthy controls and DN or post-KTx patients, were not observed (Table 4).

The concentration of endothelin-related parameters was also analyzed, taking sex into account (Tables S4 and S5 in the Supplementary Materials).

### 2.4. The Relationship Between Proteinuria and Endothelin System Parameters

**Figure 1:**
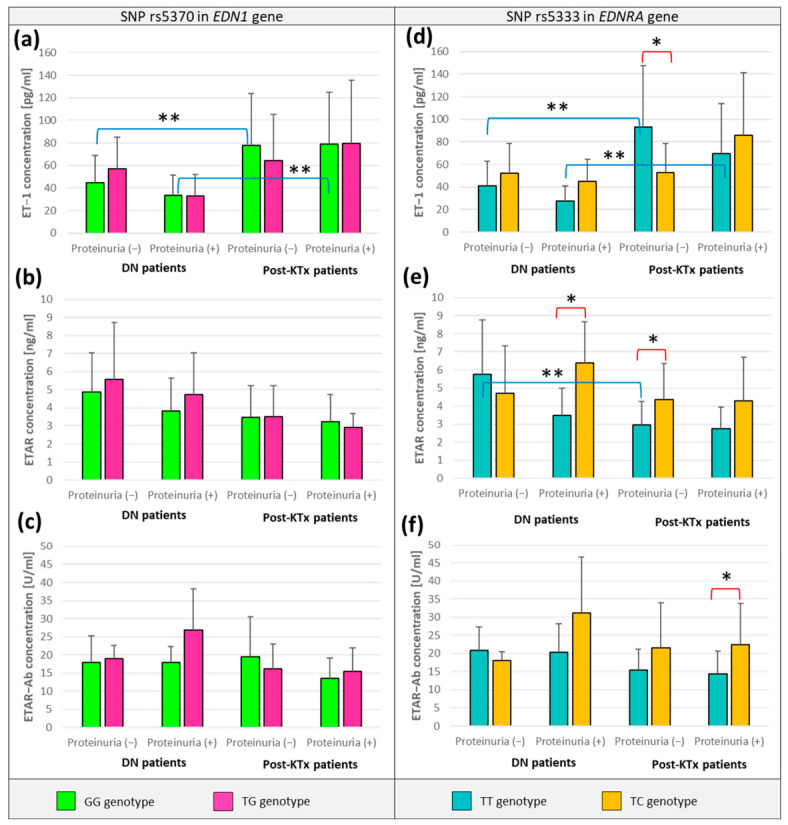
Proteinuria and endothelin system parameters. (**a**) ET-1 concentration in terms of SNP rs5370 (*EDN1* gene), (**b**) ETAR concentration in terms of SNP rs5370 (*EDN1* gene), (**c**) ETAR-Ab concentration in terms of SNP rs5370 (*EDN1* gene), (**d**) ET-1 concentration in terms of SNP rs5333 (*EDNRA* gene), (**e**) ETAR concentration in terms of SNP rs5333 (*EDNRA* gene), (**f**) ETAR-Ab concentration in terms of SNP rs5333 (*EDNRA* gene). Note: * statistically significant difference between individuals with the TT and TC genotypes for SNP rs5333 in the *EDNRA* gene (Mann–Whitney U test); ** statistically significant difference between DN patients and post-KTx patients (Kruskal–Wallis H test).

Subgroup analyses considering proteinuria showed consistently elevated ET-1 levels in post-KTx patients, regardless of proteinuria status. In post-KTx patients with the GG genotype for SNP rs5370 in the *EDN1* gene, an increased ET-1 concentration was found compared to the DN patients. These changes were noted both in the patients with proteinuria (*p* = 0.0283) and without it (*p* = 0.0014). Moreover, these differences were not observed in the above-mentioned patients with the TG genotype for SNP rs5370 in the *EDN1* gene (Figure 1a). No changes were observed in the ETAR concentration and ETAR-Ab levels between the above-mentioned patients (Figure 1b,c).

An increased ET-1 concentration was shown in post-KTx patients compared to DN patients with the TT genotype for SNP rs5333 in the *EDNRA* gene, in which proteinuria did not occur (*p* = 0.0017). This change was accompanied by a decreased ETAR concentration in this group of post-KTx patients (*p* = 0.0004) (Figure 1d,e). However, in post-KTx patients with proteinuria, an increased ET-1 concentration was found in the individuals with the TT (*p* = 0.0106) and TC genotypes (*p* = 0.0289) compared to corresponding DN patients (Figure 1d).

Interestingly, changes in the above parameters were observed between the patients with the TT and TC genotypes for SNP rs5333 in the *EDNRA* gene. In post-KTx patients with the TC genotype in whom proteinuria did not occur, a decreased ET-1 concentration (*p* = 0.0231) accompanied by increased ETAR concentration (*p* = 0.0121) was observed. An increased ETAR concentration in DN patients with the TC genotype and proteinuria compared to corresponding DN patients with the TT genotype (*p* = 0.0093) was also noted. In this group, elevated ETAR-Ab was also observed, but this change was not statistically significant (Figure 1e). Despite the slightly increased ETAR concentration in post-KTx patients with proteinuria and TC genotype (not statistically significant), elevated ETAR-Ab was observed compared to corresponding subjects with the TT genotype (*p* = 0.0438) (Figure 1f).

### 2.5. The Correlation Coefficients and the Risk of Kidney Function Loss

The correlation between the study parameters and proteinuria was examined. It was noted that the ETAR-Ab level in the DN patients with the TT genotype for SNP rs5333 in the *EDNRA* gene was positively correlated with proteinuria. In the cases of the other examined genotypes, the associations were not statistically significant (Table S6 in the Supplementary Materials).

Logistic regression analysis revealed a sex-specific association for SNP rs5370 in the *EDN1* gene. Male carriers of the TG genotype demonstrated an increased risk of kidney function loss requiring transplantation (OR = 4.4800, *p* = 0.0488) compared to their female counterparts. In the case of the GG genotype for SNP, the risk was not statistically significant (OR = 1.4975, *p* = 0.3057). No significant associations were observed for SNP rs5333 in the *EDNRA* gene (OR = 0.9333, *p* = 0.8681; OR = 2.6790, *p* = 0.0667, respectively, for males with the TT and TC genotypes).

## 3. Discussion

The importance of ET-1 for renal homeostasis and its participation in the pathophysiological mechanisms of this organ have been studied for many years [2,24,25,26,27]. The pathophysiological role of ET-1 includes several detrimental effects in different parts of the kidney, inducing vascular injury via enhanced vasoreactivity and procoagulant activity, nephron shedding, and disruption of the podocyte cytoskeleton, leading to podocyte injury. These changes result in proteinuria, chronic inflammation, tubulointerstitial fibrosis, mesangial remodeling, and accumulation of extracellular matrices [6,28]. Many studies have shown a link between increased ET-1 concentration and the activation of transcription factors, including those dependent on ERK1/2 kinases, followed by the production of proinflammatory cytokines. In turn, this process contributes to the production of ET-1, creating a positive feedback loop [29,30,31,32]. Inflammation, along with metabolic and hemodynamic changes, plays an essential role in the development of DN [3,33,34]. Patients with type 2 diabetes mellitus (T2DM) have elevated ET-1 levels compared to nondiabetic subjects, and DN promotes an additional increase in this peptide concentration in the blood [35,36,37]. These findings are consistent with the results obtained in this study. The present study demonstrates a pronounced dysregulation of the endothelin system in patients with diabetic nephropathy. Importantly, the highest ET-1 concentrations were found in post-transplant patients, indicating that kidney transplantation does not normalize endothelin system activity and may even be associated with its further activation. The elevation of ET-1 concentration observed in our cohort, particularly in post-KTx patients, may reflect ongoing endothelial dysfunction related to chronic inflammatory processes, ischemia–reperfusion injury, and immunosuppressive therapy, as suggested in other studies [38,39]. Studies indicate that cyclosporine, tacrolimus, or everolimus administration can cause endothelial dysfunction and contribute to increased plasma ET-1 levels [40,41,42,43]. Although immunosuppressive drugs are known to cause endothelial injury, inflammation, and dysregulation of vascular smooth muscle cell proliferation, the precise mechanism leading to the development of transplant vasculopathy is not yet fully understood [44]. However, it has been shown that increasing ET-1 levels after transplantation may play a significant role in the development of post-transplant complications, including organ ischemia and chronic graft rejection [6,38,45].

Endothelin-1 is a key mediator of glomerular and tubulointerstitial injury. ET-1 promotes vasoconstriction, increases glomerular permeability, and stimulates inflammatory and fibrotic pathways, ultimately contributing to proteinuria and progressive renal damage. This pathological progression is primarily attributed to a functional imbalance, characterized by the overactivation of ETAR relative to ETBR [11]. It was demonstrated that ETAR is elevated in glomerular endothelial cells of patients with focal segmental glomerulosclerosis and associated with podocyte damage and glomerular oxidative stress [11]. The study confirmed that diabetes is associated with elevated ET-1 and enhanced renal expression of ETAR [25]. The literature data are consistent with the results presented in this study, which indicate increased ETAR levels in DN patients compared to healthy controls. Moreover, the prolonged significant increase in the ET-1 concentration in post-KTx patients, due to existing diabetes and immunosuppressive drugs, can contribute to a compensatory decrease in ETAR concentration. Other studies noted that prolonged exposure to increased ET-1 concentration leads to a dose-dependent decrease in the mRNA level of ETAR [46,47].

In humans, the endothelial system may be influenced by sex [5]. Sexual dimorphism of this system can manifest at multiple levels. These include the difference in pre-pro-ET-1 expression, as well as ET-1 concentrations in plasma and tissues, the density and function of ETAR and ETBR, the physiological effects of ET-1 on the circulatory system and kidneys, and differential responses to the antagonists of these receptors [5,48]. There is evidence indicating a role of female hormones in lowering circulating ET-1 in females compared with age-matched males [49,50]. ET-1 synthesis in the vascular endothelium is attenuated in the presence of estrogens and progesterone, which can also contribute to greater endothelial ETBR expression and increase clearance of circulating ET-1 [50]. However, studies from the last decade indicate a lack of consensus on the sexual dimorphism of ET-1 concentration. Hellgren et al. [2] found slightly higher plasma ET-1 concentrations in females than in males. It is contrary to the findings of Bossard et al. [51]. Some studies have not shown the influence of age-related hormonal changes on ET-1 and ETAR expression or their concentrations [52,53]. Our study has not found an influence of sex on ET-1 and ETAR concentrations in the study groups. Moreover, the analysis of results in terms of sex showed no difference between DN and post-KTx patients in ETAR concentration in female and male subjects. This allows us to conclude that the ongoing disease process can diminish the influence of sex and age differences between the study groups on the endothelial system, as reported by other researchers [50,54].

Our earlier studies proved that the presence of non-HLA antibodies, including ETAR-Ab, is associated with worse graft function in the post-KTx patients and graft loss [55,56]. Therefore, an important point of this study was to determine ETAR-Ab. The significant reduction in ETAR-Ab levels in transplant recipients was observed, which is consistent with the findings of Senev et al. [57]. The decrease in ETAR-Ab observed in our study may be related to immunosuppressive therapy or altered immune responses following transplantation [58]. The mechanisms responsible for the reduced circulating ETAR-Ab levels observed in post-KTx recipients remain uncertain. In kidney transplant recipients, one plausible explanation is the effect of maintenance immunosuppression, which may suppress B-cell activation, plasma cell function, and de novo autoantibody production [59,60,61,62]. However, reduced plasma antibody levels do not necessarily indicate the absence of pathogenic antibody activity. ETAR-Ab may bind to ETAR expressed on endothelial, vascular smooth muscle, or renal cells, leading to partial removal from the circulating compartment [63]. Antibody binding to tissue targets, formation of immune complexes, or redistribution into vascular and intrarenal compartments may therefore contribute to lower measurable plasma concentrations [64]. In DN patients, chronic metabolic inflammation, immune dysregulation associated with diabetes and CKD, and possible antibody consumption may also influence circulating ETAR-Ab levels [65]. Another potential explanation is the development of disease- or treatment-related immune tolerance, although this cannot be directly inferred from the present data [66]. Because our study was cross-sectional and did not assess immune complexes, tissue-bound antibodies, B-cell subsets, or longitudinal antibody kinetics, we cannot determine whether the observed reduction reflects decreased antibody production, increased antibody consumption, tissue compartmentalization, or immunological tolerance. This issue requires further mechanistic and longitudinal studies.

The study population was divided in terms of SNPs relevant to ET-1 and ETAR (rs5370 and rs5333). It can implicate the individual differences in the functioning of the endothelial system, as genetic variability in endothelial pathways has been associated with DN [67]. In the male DN patients described in this paper, an increased ET-1 concentration in individuals with the TG genotype compared to those with the GG genotype (SNP rs5370 in the *EDN1* gene) was found. In contrast, in female patients, no difference in this parameter was shown. Moreover, in post-KTx males, an elevated ETAR concentration was also found. An increased ET-1 and ETAR can contribute to the deterioration of kidney function as reported in other studies [24,26,27,68,69]. It can explain the elevated risk of loss of kidney function resulting in KTx (nearly 4.5-fold higher) in male DN patients with the TG genotype compared to female patients, which was found in our study. Interestingly, this association was not observed in patients with the GG genotype. Similar results have been reported by other researchers, who have highlighted the impact of SNP rs5370 on the development of nephropathy in patients with diabetes [70]. The data presented by Ahmed et al. [70] indicate a significantly higher incidence of nephropathy among patients with TT/TG genotypes compared to patients with the GG genotype. However, it is worth noting that their study, although conducted on a Caucasian population, differs from our research in terms of the ethnic group studied. Hence, our findings fill the research gap concerning the influence of SNP rs5370 in the *EDN1* gene on the complications of nephropathy in patients with diabetes because the literature data on this subject are limited. There are numerous studies on SNP rs5370, but they concern other disease entities. It was also shown that the T allele occurrence of SNP rs5370 can be regarded as a risk factor for many diseases, e.g., coronary atherosclerosis, large artery stroke, and ischemic stroke [16,19,21,71]. However, the findings of studies dealing with nephrotic syndrome are contrary to our own. Abdul-Maksoud et al. [72] indicate that the GG genotype at the rs5370 locus of the *EDN1* gene may be associated with an increased risk of this disease. However, Hashemi et al. [17] did not find this association.

As mentioned above, the functional responses to ET-1 are mediated by ETAR and ETBR. A shift in the ratio of ETAR:ETBR expression is observed in many pathophysiological processes [5]. Therefore, an important goal of our study was to evaluate SNP rs5333 in the *EDNRA* gene and its impact on the examined parameters. The analysis of the results in terms of SNP rs5333 in the *EDNRA* gene indicated a positive correlation between ETAR-Ab concentration and proteinuria in DN patients with the TT genotype, suggesting that these patients may be at greater risk of kidney damage than patients with the TC genotype, resulting from antibody-mediated damage to the glomerular barrier. However, further analysis showed that the relationship between significantly increased ET-1 concentration and decreased ETAR concentration is apparent only in post-KTx patients with the TT genotype. This might indicate that, in this group of patients, the above-described compensatory relationship between ET-1 and ETAR is activated. Additionally, our research showed that this mechanism is prevalent in post-KTx patients with the TT genotype, in whom proteinuria did not occur. In contrast, in the individuals with the TC genotype, the above-mentioned changes in ET-1 and ETAR concentrations were not found. Moreover, higher ET-1 and ETAR concentrations were found in healthy males with this genotype compared to females. Additionally, DN patients with the TC genotype and proteinuria had a slightly increased ET-1 concentration, which was accompanied by significantly increased ETAR concentration and elevated ETAR-Ab (but not statistically significant). Similarly, in post-KTx patients with the TC genotype and proteinuria, slightly increased ET-1 and ETAR concentrations resulting in a significant increase in ETAR-Ab concentration were found. It was noted that ETAR-Ab can activate the target receptors and affect signaling pathways [13]. Their binding to ETAR results in similar effects to those triggered by natural ligands, including vasoconstriction, extracellular matrix remodeling, and proinflammatory cascades [73]. It was reported that higher ETAR-Ab concentrations are a risk factor for worse kidney function [65,74]. A previous study indicated that the blockade of ETAR diminishes proteinuria in patients with IgA nephropathy and causes renoprotection [75]. Atrasentan and avosentan, selective endothelin A receptor antagonists, contributed to reduced albuminuria in patients with diabetic nephropathy [76,77,78]. These data support a potential role for selective endothelin receptor antagonists in protecting renal function in patients with type 2 diabetes who are at high risk of developing end-stage kidney disease [77]. Moreover, our findings suggest that selective endothelin receptor antagonists could have therapeutic benefits, especially in patients with the TC genotype (SNP rs5333) and proteinuria, in whom the influence of elevated ET-1 concentration on decreased ETAR expression is not observed and can contribute to the rapid complications of nephropathy.

SNP rs5333 in the *EDNRA* gene has been studied in many diseases, including ischemic stroke, lung cancer, obesity, and intracerebral hemorrhage [21,79,80,81]. One study investigated this topic in patients with idiopathic nephrotic syndrome; however, the above-mentioned polymorphism in the *EDNRA* gene and its effect on the ET-1–ETAR system have not been studied in CKD patients so far [20]. There are also no studies regarding SNP rs5333 in the *EDNRA* gene in a Caucasian population. Therefore, the limited knowledge does not allow us to compare our results with those of other researchers. This highlights the novelty of the topic, especially in the context of drugs recently approved in Europe that are selective endothelin receptor antagonists.

Our study supports a model in which the persistent activation of the endothelin system is a key feature of CKD and post-transplant status. An important finding is the influence of genetic factors (SNPs) and sex on the endothelial system function. These findings highlight the potential clinical relevance of endothelin-related biomarkers and provide further rationale for therapeutic strategies targeting the endothelin pathway in patients with chronic kidney disease. Several limitations of this study should be acknowledged. The relatively small sample size, particularly genetic subgroups, may limit the statistical power to detect subtle associations (the statistical power to detect associations with TT for rs5370 and CC for rs5333 genotypes is extremely low). The cross-sectional design precludes assessment of causal relationships and long-term outcomes. Additionally, the lack of longitudinal follow-up data limits the evaluation of endothelin-related biomarkers as predictors of disease progression. Another significant limitation of this study is the lack of comparability between the study groups based on age, as the healthy subjects were significantly younger than the patients in the other two groups (DN group and post-KTx group). ET-1 concentrations may increase with age, so physiological factors may also have influenced the observed associations. Therefore, when assessing the differences between the studied groups, the influence of age, not just the disease itself, should be considered. Future studies should include an age-matched population, which would allow for better determination of the independent association between ET-1 and the development of DN or post-transplant status. Finally, due to limited literature data concerning Caucasian populations, our findings should be interpreted with caution and require confirmation in future research.

## 4. Materials and Methods

### 4.1. Study Population and Sample Collection

This study included 231 participants, who were divided into three groups: controls (*N* = 50) and two groups with chronic kidney disease (CKD)—a diabetic nephropathy group (DN, *N* = 83) and a diabetic nephropathy group after kidney transplantation (post-KTx, *N* = 100). All participants were informed in detail about the purpose and course of the study and gave their written consent to participate in it. The Bioethics Committee at the Medical University of Wroclaw approved the collection of biological material for research purposes (KB 835/2021) and the conduction of this study (No. KB 21/2026).

Biological material was obtained from venous blood collected in two types of tubes. Serum was collected using tubes with a clotting activator (cat. No. BD 368815, Becton Dickinson, Franklin Lakes, NJ, USA), while the plasma and buffy coat were collected using tubes containing EDTA (cat. No. BD 367864, Becton Dickinson, Franklin Lakes, NJ, USA). Genomic DNA was isolated from the buffy coat using a commercial DNA isolation kit (Syngen Blood/Cell DNA Mini Kit, cat. No. SY221012, Syngen Biotech, Wroclaw, Poland).

Control blood samples were obtained from Łukasiewicz PORT—Polish Center for Technology Development. The control group consisted of healthy individuals, in whom the following conditions were excluded: diabetes and diabetic kidney disease, cardiovascular disease, liver dysfunction, inflammatory conditions, and cancer. In addition, controls did not use any medications or dietary supplements for at least 6 months prior to blood sampling.

The other two groups included patients under the care of the Department of Nephrology, Transplantation Medicine and Internal Diseases, Institute of Internal Diseases at Wroclaw Medical University. The inclusion criteria were diagnosis of diabetes and one of the following: albuminuria, proteinuria, or elevated creatinine levels. The exclusion criterion was kidney damage of unknown etiology, evaluated on the basis of imaging studies. The post-KTx patients were subjected to immunosuppressive treatment. The vast majority of patients were taking a triple-drug regimen: tacrolimus administered every 12 or 24 h, mycophenolate mofetil administered every 12 h, and steroid therapy administered every 24 h. The group of patients using other immunosuppressive regimens—dual therapy without mycophenolate mofetil, with the use of cyclosporine or azathioprine—constituted a significantly smaller group. Additionally, all participants completed a questionnaire covering demographic and clinical data, such as age, sex, body weight, height, comorbidities, smoking, alcohol consumption, and medications and dietary supplements used.

The characteristics of the study groups are presented in Table 5. It contains data on age, sex, smoking, body mass index (BMI), glucose concentration, creatinine, estimated glomerular filtration rate (eGFR), and C-reactive protein (CRP) concentration.

### 4.2. Methods

#### 4.2.1. Genotyping Analysis of rs5370 and rs5333 Single Nucleotide Polymorphisms (SNPs)

Genotyping of SNP rs5370 in the *EDN1* gene and SNP rs5333 in the *EDNRA* gene was performed using polymerase chain reaction–restriction fragment length polymorphism (PCR-RFLP). Primers were designed using Primer-BLAST 2.5.0 (National Center for Biotechnology Information, Bethesda, MD, USA) based on gene sequences obtained from GenBank (National Center for Biotechnology Information, Bethesda, MD, USA). Detailed information on the primer sequences and reaction conditions is provided in Table S7 in the Supplementary Materials. The following restriction enzymes were used for genotyping the polymorphisms: Cac8I (SNP rs5370; cat. No. R0579L, New England Biolabs, Ipswich, MA, USA) and BspTi (SNP rs5333; LOT: ER0925, Thermo Fisher Scientific, Waltham, MA, USA). Digested DNA fragments were visualized on a 3% agarose gel stained with Green DNA Gel Stain (cat. No. SY521031, Syngen Biotech, Wroclaw, Poland).

#### 4.2.2. Assessment of Biochemical and Endothelin-Related Parameters

Plasma/serum glucose and creatinine concentrations, as well as proteinuria, were measured in the hospital laboratory during routine patient visits. eGFR was calculated using the Modification of Diet in Renal Disease (MDRD) equation.

Plasma endothelin-1 (ET-1) concentration was measured using the Human Endothelin-1 ELISA Kit (cat. no.: HUFI00466, Assay Genie, Dublin, Ireland). Plasma endothelin receptor type A (ETA) concentration was measured using the Human ETRA/Endothelin A Receptor ELISA Kit (cat. no.: HUFI02431, Assay Genie, Dublin, Ireland). In turn, the level of antibodies against ETA in blood plasma was determined using the EIA for Quantitative Determination of anti-Endothelin Receptor A (ETA)-Antibodies test (cat. no.: 12100, CellTrend, Luckenwalde, Germany). Appropriate negative controls were included in the ELISA assays to validate the method. For the first two tests, the controls were blank samples, and for the third test, it was a negative control included in the kit.

#### 4.2.3. Statistical Analysis

Statistical analyses were performed using STATISTICA software version 14.1.0.4 (StatSoft Polska Sp. z o.o., Krakow, Poland), licensed by the Wroclaw Medical University. The normality of the data distribution was evaluated using the Shapiro–Wilk test.

The differences between the two groups were analyzed using Student’s *t*-test for variables with a normal distribution or the Mann–Whitney U test when the normality assumptions were not met. The Kruskal–Wallis H test with Dunn’s post hoc test was used for comparisons between three or more groups. For multiple comparisons, the correction test based on the asymptotic normal distribution for mean ranks was used.

The frequency of genotypes was compared using the χ2 test and Fisher’s exact test. To assess the association between polymorphism genotypes, the risk of developing diabetic kidney disease, and the need for renal replacement therapy, a logistic regression analysis was performed. The results were presented as odds ratios (OR) with 95% confidence intervals (CI). To verify correlations between the examined parameters, the Spearman correlation was used. The statistical significance level was set at *p* < 0.05. The data are presented as mean ± standard deviation.

## 5. Conclusions

1. Endothelin system dysregulation (reflected by elevated plasma ET-1 and ETAR concentrations) is a prominent and persistent feature of CKD and remains evident after kidney transplantation.
2. In contrast to the increased levels of endothelin system proteins, ETAR-Ab levels are significantly lowered in kidney transplant recipients compared to healthy individuals. This occurs in both sexes and may be related to immunological processes or post-transplant therapy.
3. SNP rs5370 in the *EDN1* gene has an influence on the interindividual differences in endothelial system function. Males with the TG genotype have a nearly 4.5-fold higher risk of renal replacement therapy compared to females with the same genotype.
4. SNP rs5333 in the *EDNRA* gene differentiates the physiological changes at the receptor level:
  - The blood of kidney transplant recipients with the TT genotype (without proteinuria) showed a unique relationship—an increase in ET-1 was accompanied by a decrease in ETAR concentration, which may reflect the action of a compensatory mechanism;
  - The patients with the TC genotype (particularly those with proteinuria) tended to have elevated concentrations of ETAR and ETAR-Ab, which may exacerbate renal pathology. This suggests that such patients may particularly benefit from the use of selective endothelin receptor antagonists to protect renal function.

## Figures and Tables

**Table 1:** The concentration of endothelial-related parameters in the study groups.

		CKD Patients	
Parameters	Healthy Controls	DN Patients	Post-KTx Patients
ET-1 [pg/mL]	5.2 ± 1.7	43.6 ± 26.1 ^1^	76.2 ± 48.9 ^1,2^
ETAR [ng/mL]	1.6 ± 1.0	5.0 ± 2.7 ^1^	4.1 ± 3.0 ^1,2^
ETAR-Ab [U/mL]	27.9 ± 9.0	21.4 ± 9.5 ^1^	18.2 ± 9.9 ^1^

Note: ^1^ statistically significant difference compared to healthy controls (Kruskal–Wallis H test); ^2^ statistically significant difference compared to DN patients (Kruskal–Wallis H test).

**Table 2:** Genotype distribution across study groups.

**SNP rs5370** **(*EDN1*)**	**Genotype**	**CKD Patients**	**Healthy Controls**	**OR (95% CI)**	***p*** **-Value**
**Codominant**	G/G	122 (66.7%)	33 (71.7%)	1.00	0.62
	T/G	52 (28.4%)	10 (21.7%)	1.41 (0.65–3.06)	
	T/T	9 (4.9%)	3 (6.5%)	0.81 (0.21–3.17)	
**Dominant**	G/G	122 (66.7%)	33 (71.7%)	1.00	0.51
	T/G-T/T	61 (33.3%)	13 (28.3%)	1.27 (0.62–2.59)	
**Recessive**	G/G-T/G	174 (95.1%)	43 (93.5%)	1.00	0.67
	T/T	9 (4.9%)	3 (6.5%)	0.74 (0.19–2.86)	
**Overdominant**	G/G-T/T	131 (71.6%)	36 (78.3%)	1.00	0.35
	T/G	52 (28.4%)	10 (21.7%)	1.43 (0.66–3.09)	
**SNP rs5333** **(*EDNRA*)**	**Genotype**	**CKD Patients**	**Healthy Controls**	**OR (95% CI)**	***p*** **-Value**
**Codominant**	T/T	116 (64.8%)	29 (58%)	1.00	0.6
	T/C	58 (32.4%)	20 (40%)	0.72 (0.38–1.39)	
	C/C	5 (2.8%)	1 (2%)	1.25 (0.14–11.12)	
**Dominant**	T/T	116 (64.8%)	29 (58%)	1.00	0.38
	T/C-C/C	63 (35.2%)	21 (42%)	0.75 (0.40–1.42)	
**Recessive**	T/T-T/C	174 (97.2%)	49 (98%)	1.00	0.75
	C/C	5 (2.8%)	1 (2%)	1.41 (0.16–12.34)	
**Overdominant**	T/T-C/C	121 (67.6%)	30 (60%)	1.00	0.32
	T/C	58 (32.4%)	20 (40%)	0.72 (0.38–1.37)	

**Table 3:** Endothelial-related parameters in relation to genetic variants for SNP rs5370 in the *EDN1* gene.

Parameter	Genotype (SNP rs5370 in *EDN1* Gene)	Healthy Controls	DN Patients	Post-KTx Patients
ET-1 [pg/mL]	GG	5.2 ± 1.4	40.4 ± 22.9 ^1^	77.4 ± 45.6 ^1,2^
	TG	5.0 ± 2.3	56.6 ± 30.8 ^1^	69.9 ± 50.4 ^1^
ETAR [ng/mL]	GG	1.6 ± 0.9	4.7 ± 2.5 ^1^	4.6 ± 4.9 ^1^
	TG	1.4 ± 1.1	5.5 ± 3.0 ^1^	4.2 ± 2.8 ^1^
ETAR-Ab [U/mL]	GG	27.4 ± 8.1	20.4 ± 10.0 ^1^	18.9 ± 11.0 ^1^
	TG	26.2 ± 12.8	21.9 ± 7.2	16.8 ± 7.3

Note: ^1^ statistically significant difference compared to healthy controls (Kruskal–Wallis H test), ^2^ statistically significant difference compared to DN patients (Kruskal–Wallis H test).

**Table 4:** Endothelial-related parameters in relation to genetic variants for SNP rs5333 in the *EDNRA* gene.

Parameter	Genotype (SNP rs5333 in *EDNRA* Gene)	Healthy Controls	DN Patients	Post-KTx Patients
ET-1 [pg/mL]	TT	5.3 ± 1.8	39.0 ± 25.7 ^1^	80.5 ± 4.8 ^1,2^
	TC	5.0 ± 1.6	48.9 ± 24.6 ^1^	72.7 ± 47.0 ^1^
ETAR [ng/mL]	TT	1.7 ± 0.9	4.9 ± 2.8 ^1^	4.2 ± 4.8 ^1,2^
	TC	1.6 ± 1.1	5.2 ± 2.6 ^1^	5.0 ± 3.8 ^1^
ETAR-Ab [U/mL]	TT	26.0 ± 7.9	21.6 ± 7.9	16.6 ± 8.7 ^1,2^
	TC	30.7 ± 10.2	22.5 ± 13.2	20.1 ± 11.9 ^1^

Note: ^1^ statistically significant difference compared to healthy controls (Kruskal–Wallis H test), ^2^ statistically significant difference compared to DN patients (Kruskal–Wallis H test).

**Table 5:** Characteristics of the study groups.

Parameter	Healthy Subjects (*N* = 50)		DN Group (*N* = 83)		Post-KTx Group (*N* = 100)		*p*
	M (*N* = 21)	W (*N* = 29)	M (*N* = 43)	W (*N* = 40)	M (*N* = 51)	W (*N* = 49)	
Age [years]	35.76 ± 11.17	35.07 ± 12.05	68.51 ± 11.79 ^1^	72.08 ± 10.39 ^2^	61.38 ± 9.09 ^1^	59.72 ± 12.05 ^2,3^	<0.001
Smoking [No/Yes]	No: 16 Yes: 5	No: 27 Yes: 2	No: 37 Yes: 6	No: 38 Yes: 2	No: 48 Yes: 3	No: 46 Yes: 3	0.134
BMI [kg/m^2^]	25.25 ± 3.38	22.81 ± 3.02	30.13 ± 4.78 ^1^	30.19 ± 6.18 ^2^	28.01 ± 4.33 ^1^	26.10 ± 5.01 ^3^	<0.001
Glucose [mg/dL]	86.11 ± 7.16	84.91 ± 5.81	143.07 ± 53.42 ^1^	147.37 ± 48.65 ^2^	148.64 ± 44.98 ^1^	145.52 ± 55.79 ^2^	<0.001
Creatinine [mg/dL]	–	–	1.67 ± 0.73	1.51 ± 0.90	1.47 ± 0.45	1.45 ± 0.64	1.000
eGFR [mL/min/1.73 m^2^]	–	–	55.29 ± 36.69	44.56 ± 16.53	56.64 ± 16.01	46.72 ± 18.02	1.000

Values are given as mean value ± standard deviation. M—men; W—women; ^1^ statistically significant difference compared to healthy males; ^2^ statistically significant difference compared to healthy males and females; ^3^ statistically significant difference compared to DN patients (females). The Kruskal–Wallis test with Dunn’s post hoc test was used to compare variables such as age, BMI, glucose, creatinine, and eGFR. The χ2 test was used to compare qualitative variables (smoking).

## Data Availability

The data presented in this study are available on request from the corresponding author due to ethical restrictions.

## Supplementary Materials

The following supporting information can be downloaded at: https://www.mdpi.com/article/10.3390/ijms27135647/s1.
