# Supplementary material for: Activation of the Endothelin System in Chronic Kidney Disease and Kidney Transplant Recipients—Implications for Disease Progression
Source: Int J Mol Sci. 2026 Jun 23;27(13):5647. doi: 10.3390/ijms27135647 (PMC13360933; doi:10.3390/ijms27135647)
Supplement: Supplementary file 1 [file ijms-27-05647-s001.zip › ijms-4360169-supplementary.pdf]

Table S1. The concentration of endothelin system parameters in terms of sex.

Parameter	Healthy Controls		CKD Patients			
			DN Patients		Post-KTx Patients	
	Women	Men	Women	Men	Women	Men
ET-1 [pg/mL]	5.0 ± 1.6	6.0 ± 1.9	43.5 ± 25.6 [1]	43.8 ± 27.0 [2]	76.7 ± 46.3 [1,3]	76.2 ± 51.7 [2,4]
ETAR [ng/mL]	1.4 ± 0.9	2.0 ± 1.0	5.2 ± 2.7 [1]	4.8 ± 2.7 [2]	4.4 ± 3.6 [1]	3.8 ± 2.2 [2]
ETAR-Ab [U/mL]	27.6 ± 9.4	28.4 ± 8.7	22.9 ± 9.7	20.2 ± 9.4 [2]	17.9 ± 10.1 [1]	18.4 ± 9.9 [2]

[1] statistically significant difference compared to healthy women (Kruskal-Wallis H test), [2] statistically significant difference compared to healthy men (Kruskal-Wallis H test), [3] statistically significant difference compared to women with DN (Kruskal-Wallis H test), [4] statistically significant difference compared to men with DN (Kruskal-Wallis H test)

The increases in ET-1 and ETAR concentrations in group of patients with DN and post-KTx were found in both, women ($p<0.0001$ for comparisons of ET-1 and ETAR concentration) and men (ET-1: $p<0.0001$ for all comparisons; ETAR: $p<0.0001$ for comparison DN patients and heathy subjects, $p=0.0038$ for comparisons post-KTx patients and heathy subjects) (Table S2). Moreover, ET-1 concentration in the blood of kidney transplant recipients was increased compared to DN groups, in both women and men ($p=0.0409$ and $p=0.0412$, respectively). The decrease in ETAR-Ab levels in post-KTx patients compared to heathy subjects was also observed in both sexes ($p<0.0001$ and $p=0.0002$ for women and men, respectively). A lower ETAR-Ab level in the blood of men with DN compared to heathy subjects ($p=0.0288$) was also shown (Table S2).

Figure S1. Electrophoresis pattern of SNP rs5370 in the *EDN1* gene.

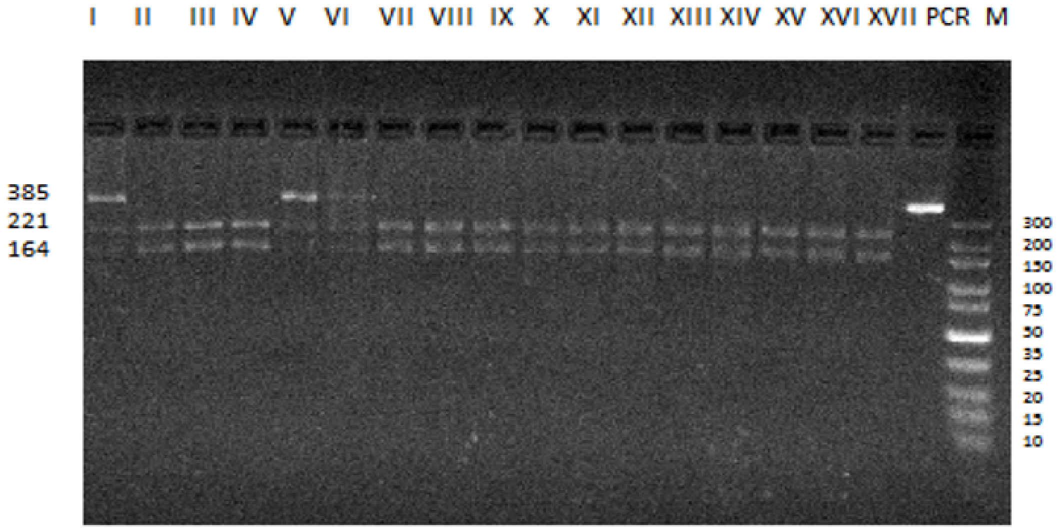

I,V,VI – T/G (164-, 221- and 385-bp fragments); II-IV, VII-XVII – G/G (164-,221-bp fragments); M- marker ladder (10-300 bp), PCR – undigested PCR product (control); Numbers are in base pair (bp).

Figure S2. Electrophoresis pattern of SNP rs5333 in the *EDNRA* gene.

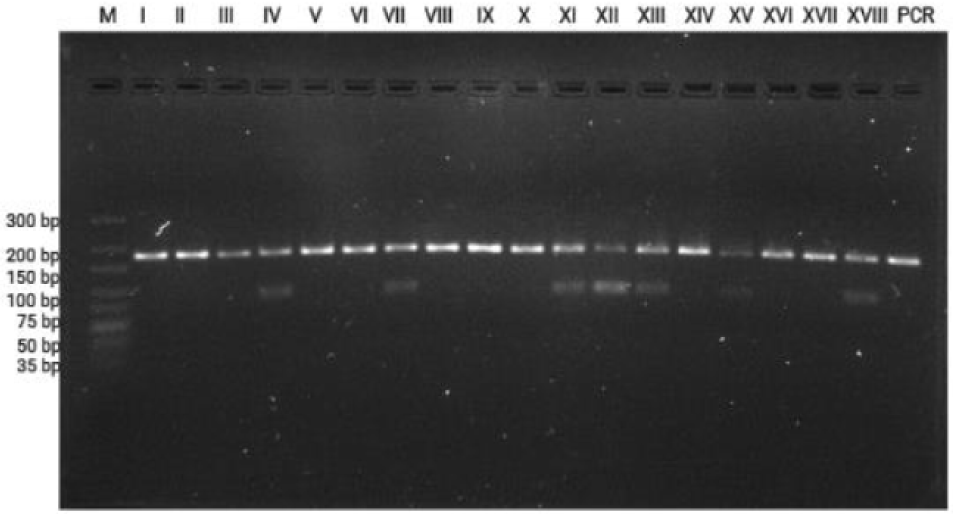

I, II, III, V, VI, VIII, IX, X, XIV, XVI, XVII – T/T (173 bp fragment); IV, VII, XI, XII, XIII, XV, XVIII – T/C (173-, 89-, 84-bp fragments); M- marker ladder (10-300 bp), PCR – undigested PCR product (control); Numbers are in base pair (bp).

Table S2. Genotype distribution in the group of DN patients and healthy controls.

SNP rs5370 (*EDN1*)	Genotype	DN Patients	Healthy Controls	OR (95% CI)	p- Value
**Codominant**	G/G	53 (63.9%)	33 (71.7%)	1.00	0.58
	T/G	25 (30.1%)	10 (21.7%)	0.64 (0.27-1.51)	
	T/T	5 (6%)	3 (6.5%)	0.96 (0.22-4.30)	
**Dominant**	G/G	53 (63.9%)	33 (71.7%)	1.00	0.36
	T/G-T/T	30 (36.1%)	13 (28.3%)	0.70 (0.32-1.52)	
**Recessive**	G/G-T/G	78 (94%)	43 (93.5%)	1.00	0.91
	T/T	5 (6 %)	3 (6.5%)	1.09 (0.25-4.78)	
**Overdominant**	G/G -T/T	58 (69.9%)	36 (78.3%)	1.00	0.30
	T/G	25 (30.1%)	10 (21.7%)	0.64 (0.28-1.50)	
**SNP rs5333 (*EDNRA*)**	**Genotype**	**DN patients**	**Healthy Controls**	**OR (95% CI)**	**p- Value**
**Codominant**	T/T	55 (67.9%)	29 (58%)	1.00	0.23
	T/C	26 (32.1%)	20 (40%)	1.46 (0.70-3.05)	
	C/C	0 (0 %)	1 (2%)	NA (0.0-NA)	
**Dominant**	T/T	55 (67.9%)	29 (58%)	1.00	0.25
	T/C-C/C	26 (32.1%)	21 (42%)	0.75 (0.40-1.42)	
**Recessive**	T/T-T/C	81 (100%)	49 (98%)	1.00	0.16
	C/C	0 (0 %)	1 (2%)	1.41 (0.16-12.34)	
**Overdominant**	T/T-C/C	55 (67.9%)	30 (60%)	1.00	0.36
	T/C	26 (32.1%)	20 (40%)	0.72 (0.38-1.37)	

Table S3. Genotype distribution in the group of post-KTx patients and healthy subjects.

SNP rs5370 (*EDN1*)	Genotype	post-KTx Patients	Healthy Controls	OR (95% CI)	p- Value
**Codominant**	G/G	69 (69%)	33 (71.7%)	1.00	0.68
	T/G	27 (27%)	10 (21.7%)	1.29 (0.56-2.98)	
	T/T	4 (4%)	3 (6.5%)	0.64 (0.13-3.01)	
**Dominant**	G/G	69 (69%)	33 (71.7%)	1.00	0.74
	T/G-T/T	31 (31%)	13 (28.3%)	1.14 (0.53-2.46)	
**Recessive**	G/G-T/G	96 (96%)	43 (93.5%)	1.00	0.52
	T/T	4 (4%)	3 (6.5%)	0.60 (0.13-2.78)	
**Overdominant**	G/G -T/T	73 (73%)	36 (78.3%)	1.00	0.49
	T/G	27 (27%)	10 (21.7%)	1.33 (0.58-3.05)	
**SNP rs5333 (*EDNRA*)**	**Genotype**	**post-KTx Patients**	**Healthy Controls**	**OR (95% CI)**	**p- Value**
**Codominant**	T/T	61 (62.2%)	29 (58%)	1.00	0.48
	T/C	32 (32.6%)	20 (40%)	0.76 (0.37-1.55)	
	C/C	5 (5.1 %)	1 (2%)	2.38 (0.27-21.28)	
**Dominant**	T/T	61 (62.2%)	29 (58%)	1.00	0.62
	T/C-C/C	37 (37.8%)	21 (42%)	0.84 (0.42-1.68)	
**Recessive**	T/T-T/C	93 (94.9%)	49 (98%)	1.00	0.34
	C/C	5 (5.1 %)	1 (2%)	2.63 (0.30-23.18)	
**Overdominant**	T/T-C/C	66 (67.3%)	30 (60%)	1.00	0.38
	T/C	32 (32.6%)	20 (40%)	0.73 (0.36-1.47)	

Table S4. The concentration of endothelial-related parameters in terms of sex and genetic variants for SNP rs5370 in the *EDN1* gene.

		**Healthy Controls**		**DN Patients**		**Post-KTx Patients**	
**Parameter**	**Genotype (SNP rs5370 in *EDN1* gene)**	Women	Men	Women	Men	Women	Men
ET-1 [pg/mL]	GG	5.1 ± 1.2	5.4 ± 1.8	45.2 ± 24.4 [1]	35.4 ± 20.7 [2, 4]	80.1 ± 46.4 [1]	75.0 ± 45.5 [2, 3]
	TG	5.2 ± 2.5	4.2 ± 2.2	45.4 ± 32.6 [1]	65.6 ± 27.6 [2]	81.0 ± 58.6 [1]	55.8 ± 35.3 [2]
ETAR [ng/mL]	GG	1.5 ± 1.0	1.8 ± 0.8	5.2 ± 2.8 [1]	4.2 ± 2.1 [2]	5.0 ± 4.0 [1]	3.5 ± 1.9 [2, 4]
	TG	1.1 ± 0.7	1.7 ± 0.7	5.5 ± 2.9 [1]	5.5 ± 3.2	3.4 ± 2.7 [1]	5.3 ± 2.7
ETAR-Ab [U/mL]	GG	27.8 ± 7.4	26.9 ± 9.1	21.2 ± 9.8	19.9 ± 10.6	18.9 ± 11.5 [1]	18.8 ± 10.7 [2]
	TG	26.3 ± 13.8	24.7 ± 0.0	23.8 ± 9.6	20.8 ± 6.5	15.9 ± 6.9	17.9 ± 8.0

[1] statistically significant difference compared to healthy women (Kruskal-Wallis H test), [2] statistically significant difference compared to healthy men (Kruskal-Wallis H test), [3] statistically significant difference compared to men with DN (Kruskal-Wallis H test), [4] $p<0.05$ compared to TG genotype (Student's t-test)

An increased ET-1 concentration was observed in the blood of women with the GG genotype (SNP rs5370 in the *EDN1* gene) with DN ($p<0.0001$) and post-KTx ($p<0.0001$) compared to healthy subjects. These changes were also shown in the group of women with the TG genotype ($p=0.0211$ and $p=0.0001$, respectively). In the group of men with the GG genotype, similarly to the appropriate group of women, an increased ET-1 concentrations in the blood of patients with DN ($p<0.0001$) and post-KTx ($p=0.0019$) compared to healthy subjects were shown. These changes were also shown in the group of men with the TG genotype ($p=0.0360$ and $p=0.0071$, respectively). Moreover, in the group of post-KTx men with the GG genotype, the statistically significant increase in ET-1 concentration compared to men with DN was found ($p=0.0106$). Interestingly, it was shown near 2-fold increase in ET-1 concentrations in the blood DN men with the TG genotype compared to appropriate group with the GG genotype ($p=0.0017$) (Table S5).

It was shown an increased ETAR concentration in the blood of women with the GG genotype with DN ($p<0.0001$) and post-KTx ($p<0.0001$) compared to healthy subjects. Similarly, these changes were also shown in the group of men with the GG genotype ($p=0.0003$ and $p=0.0092$, respectively). An increased ETAR concentration was found in the blood of women with DN ($p=0.0005$) and post-KTx ($p=0.0303$) with the TG genotype compared to healthy subjects, in

contrast to the group of men, in which these changes were not shown. However, it was shown an increase in ETAR concentrations in the blood post-KTx men with the TG genotype compared to appropriate group with the GG genotype (p=0.0157) (Table S5).

Interestingly, decreased ETAR-Ab levels in the blood of post-KTx patients compared to healthy subjects were observed in both, women (p=0.0015) and men (p=0.0101) with the GG genotype. These differences were not found in the groups of the patients with the TG genotype (Table S5).

Table S5. The concentration of endothelial-related parameters in terms of sex and genetic variants for SNP rs5333 in the *EDNRA* gene.

		**Healthy Controls**		**DN patients**		**Post-KTx patients**	
**Parameter**	**Genotype (SNP rs5333 in *EDNRA* gene)**	Women	Men	Women	Men	Women	Men
ET-1 [pg/mL]	TT	5.4 ± 1.9	5.2 ± 1.8	36.9 ± 23.2 [1]	40.9 ± 28.2 [2]	90.5 ± 54.6 [1, 3]	69.3 ± 43.3 [2]
	TC	4.5 ± 1.3 [4]	6.5 ± 1.9	52.0 ± 26.6 [1]	44.8 ± 20.1	60.0 ± 33.4 [1]	80.9 ± 53.1 [2]
ETAR [ng/mL]	TT	1.5 ± 0.9	1.8 ± 1.0	4.8 ± 2.8 [1]	5.1 ± 2.8 [2]	3.7 ± 2.4 [1]	3.5 ± 2.4 [2, 5]
	TC	1.2 ± 0.9 [4]	2.5 ± 1.1	6.0 ± 2.4 [1]	4.2 ± 2.6	6.5 ± 5.6 [1]	4.2 ± 1.8
ETAR-Ab [U/mL]	TT	24.6 ± 7.1	27.1 ± 8.7	24.6 ± 8.7 [1]	19.2 ± 6.7	15.2 ± 6.2 [1]	18.4 ± 10.8 [2]
	TC	30.3 ± 11.1	31.9 ± 8.3	20.0 ± 11.7	25.7 ± 16.1	23.8 ± 12.8	17.7 ± 9.2 [2]

[1] statistically significant difference compared to healthy women (Kruskal-Wallis H test), [2] statistically significant difference compared to healthy men (Kruskal-Wallis H test), [3] statistically significant difference compared to women with DN (Kruskal-Wallis H test), [4] statistically significant difference compared to healthy men (Student's t-test), [5] statistically significant difference compared to men with DN (Kruskal-Wallis H test).

It was shown an increased ET-1 concertation in the blood of women with the TT genotype with DN ($p=0.0014$) and post-KTx ($p<0.0001$) compared to healthy subjects. These changes were also shown in the group of men with the TT genotype ($p=0.0001$ and $p<0.0001$, respectively). Additionally, in the group of post-KTx women with this genotype, an increased ET-1 concentration compared to appropriate group of DN women was found ($p=0.0222$). In the group of patients with the TC genotype, an increased ET-1 concentrations in the blood of post-KTx women ($p<0.0001$) and men ($p=0.0011$) compared to healthy subjects were shown. Moreover, in the group of DN women with the TC genotype, an increase in the concentration of this peptide compared to appropriate group of healthy women ($p<0.0001$) was found. Interestingly, it was noted that the ET-1 concentration was increased in the blood of healthy men with the TC genotype compared to healthy women ($p=0.0263$) (Table S6).

ETAR concentration was elevated in the blood of healthy men with the TC genotype compared to healthy women ($p=0.0222$) (Table S6). It was noted a significantly increase in the ETAR concentration in the blood of post-transplant women and the women with DN compared to healthy women in both, the

individuals with the TT ($p=0.0014$ and $p<0.0001$, respectively) and TC genotypes ($p=0.0016$ and $p<0.0001$, respectively). However, in the blood of men, the changes in ETAR concentration were only visible in the case of individuals with the TT genotype. An increased ETAR concentration in the blood of post-KTx men and DN men with the TT genotype compared to appropriate group of healthy subjects were shown ($p<0.0001$ for both comparisons). Additionally, ETAR level in the blood of DN men with the TT genotypes was also increased compared to the concentration of this parameter in the blood of post-KTx men with the same genotype ($p=0.0322$) (Table S6).

An decreased ETAR-Ab in the blood of post-KTx women with the TT genotype compared to appropriate group of healthy subjects ($p=0.0005$) and DN women ($p=0.0041$) were shown. Similar differences in the level of ETAR-Ab were noted in the blood of post-KTx men compared to healthy men in both, the individuals with the TT ($p=0.0044$) and TC ($p=0.0293$) genotype (Table S6).

Table S6. Results of correlations between endothelial-related parameters and proteinuria across study groups.

SNP rs5370 in the *EDN1* gene			
**Genotype**	**Correlated parameters**	**DN Patients**	**Post-KTx Patients**
**GG genotype**	**ET-1 [pg/mL]**	r= 0.1367<br>p=0.566	r=-0.1955<br>p=0.138
	**ETAR [ng/mL]**	r=-0.0729<br>p=0.760	r=-0.1650<br>p=0.212
	**ETAR-Ab [U/mL]**	r=0.2679<br>p=0.254	r=-0.0716<br>p=0.590
**TG genotype**	**ET-1 [pg/mL]**	r=-0.2122<br>p=0.648	r=-0.0962<br>p=0.662
	**ETAR [ng/mL]**	r=-0.1387<br>p=0.767	r=-0.1547<br>p=0.481
	**ETAR-Ab [U/mL]**	r=0.1343<br>p=0.774	r=-0.1240<br>p=0.573
**SNP rs5333 in the *EDNRA* gene**			
**Genotype**	**Correlated parameters**	**DN patients**	**Post-KTx patients**
**TT genotype**	**ET-1 [pg/mL]**	r=0.2536<br>p=0.295	r=-0.1993<br>p=0.157
	**ETAR [ng/mL]**	r=-0.898<br>p=0.715	r=-0.1792<br>p=0.204
	**ETAR-Ab [U/mL]**	**r=0.4911**<br>**p=0.028**	r=-0.1198<br>p=0.397
**TC genotype**	**ET-1 [pg/mL]**	r=-0.1336<br>p=0.732	r=0.1586<br>p=0.394
	**ETAR [ng/mL]**	r=-0.2398<br>p=0.534	r=-0.2304<br>p=0.212
	**ETAR-Ab [U/mL]**	r=0.2903<br>p=0.449	r=-0.0012<br>p=0.995

Table S7. Primer sequences and PCR conditions.

SNP (*Gene*)	Primers	PCR-RFLP Conditions
rs5370 (*EDN1*)	Forward primer:<br>5′ CAG ATT CAG GTT TTG TTT GTG CCA GAT T 3′<br>Reverse primer:<br>5′ TTG GGG GAA CTC CTT AAC CTT TCT TG 3′	polymerase activation: 95 °C for 15 min<br>denaturation: 95 °C for 40 s<br>annealing: 77 °C for 35 s<br>elongation: 72 °C for 40 s<br>final elongation: 72 °C for 10 min
rs5333 (*EDNRA*)	Forward primer:<br>5′ TTT TTC TCA CTT TCC TTT AG 3′<br>Reverse primer:<br>5′ CGA GTA CAC AGG ATC ATA CC 3′	polymerase activation: 95 °C for 15 min<br>denaturation: 95 °C for 40 s<br>annealing: 54 °C for 35 s<br>elongation: 72 °C for 40 s<br>final elongation: 72 °C for 10 min
